# Supplementary material for: LC-MS and GC-MS Data Fusion Metabolomics Profiling Coupled with Multivariate Analysis for the Discrimination of Different Parts of Faustrime Fruit and Evaluation of Their Antioxidant Activity
Source: Antioxidants (Basel). 2023 Feb 24;12(3):565. doi: 10.3390/antiox12030565 (PMC10045819; doi:10.3390/antiox12030565)
Supplement: Supplementary file 1 [file antioxidants-12-00565-s001.zip › antioxidants-2223045-supplementary.pdf]

Supplementary Material

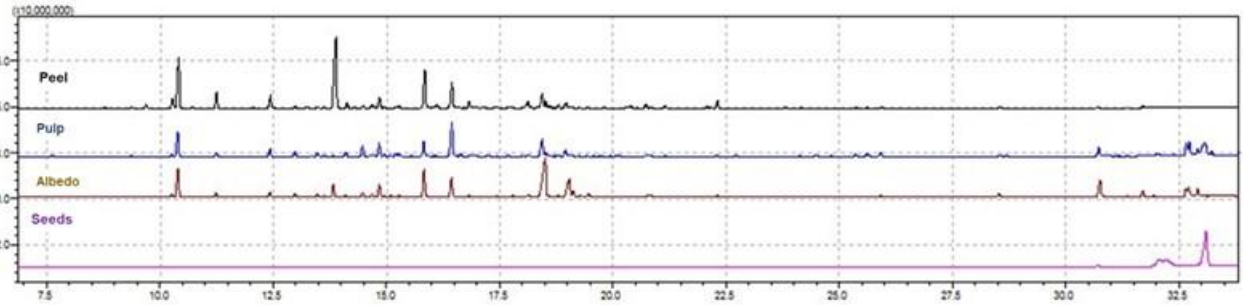

Figure S1. GC-MS Profiles of PPAS parts n-hexane extracts

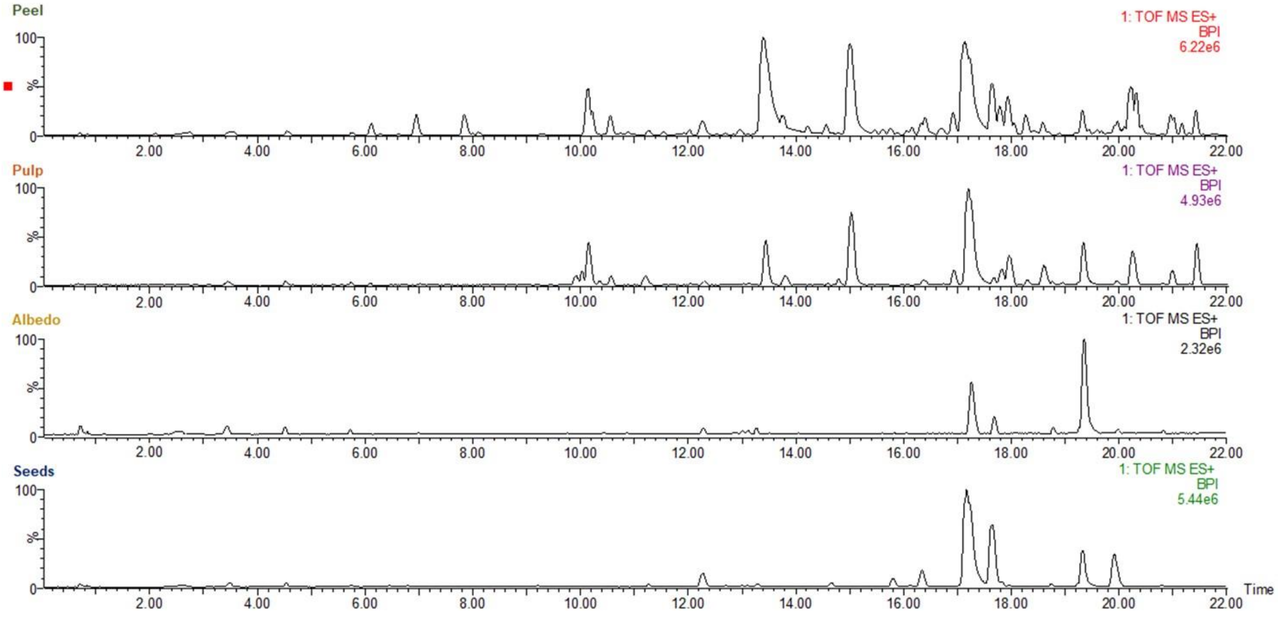

Figure S2. UPLC-HRMS profiles of PPAS parts chloroform extracts

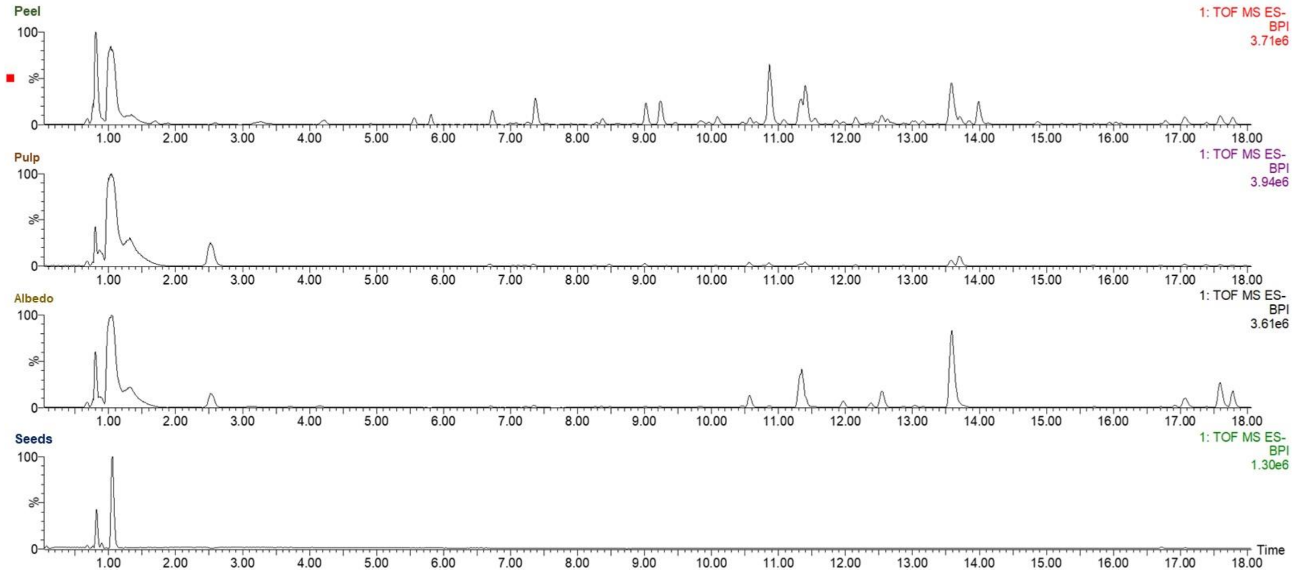

Figure S3. UPLC-HRMS profiles of PPAS parts methanol/water (50% v/v) extracts

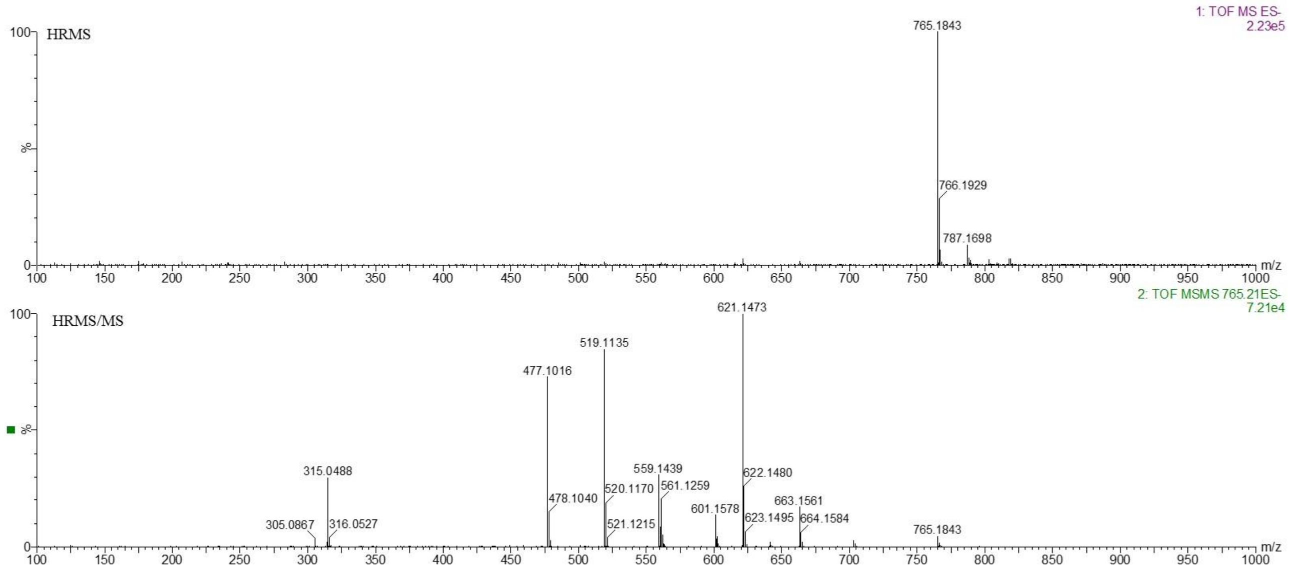

Figure S4. MS/HRMS spectra of compound 91

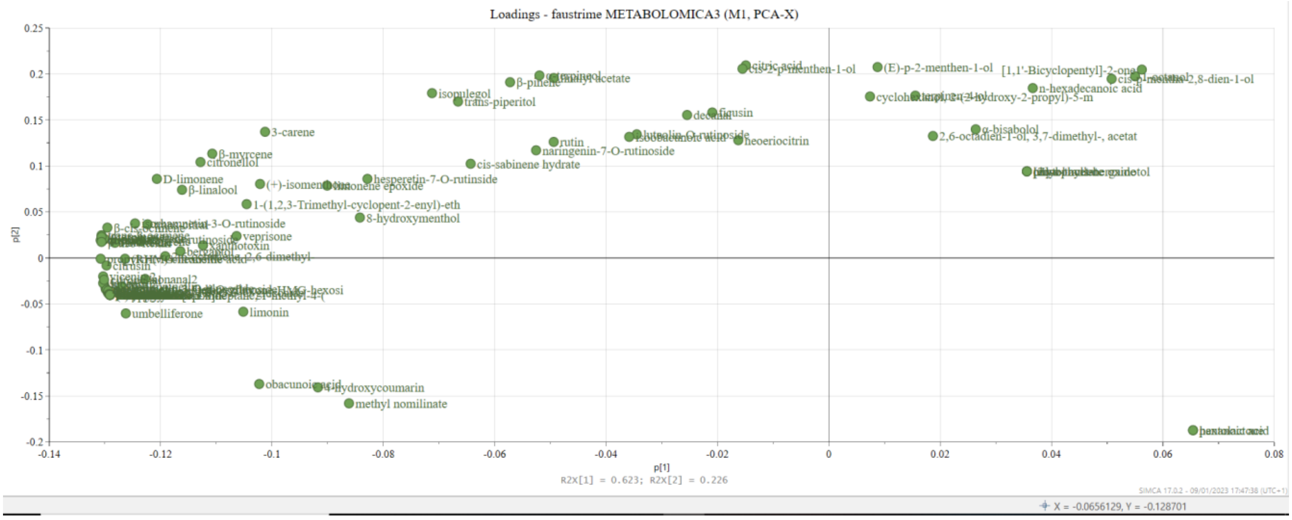

Figure S5. PCA Loading Plot

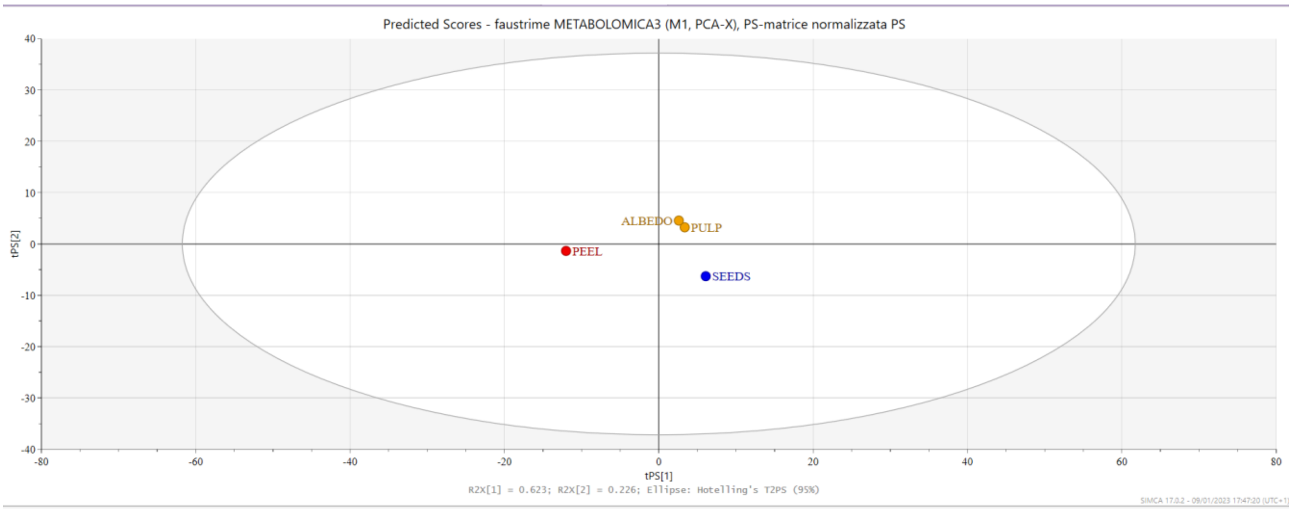

Figure S6. PCA Score Plot

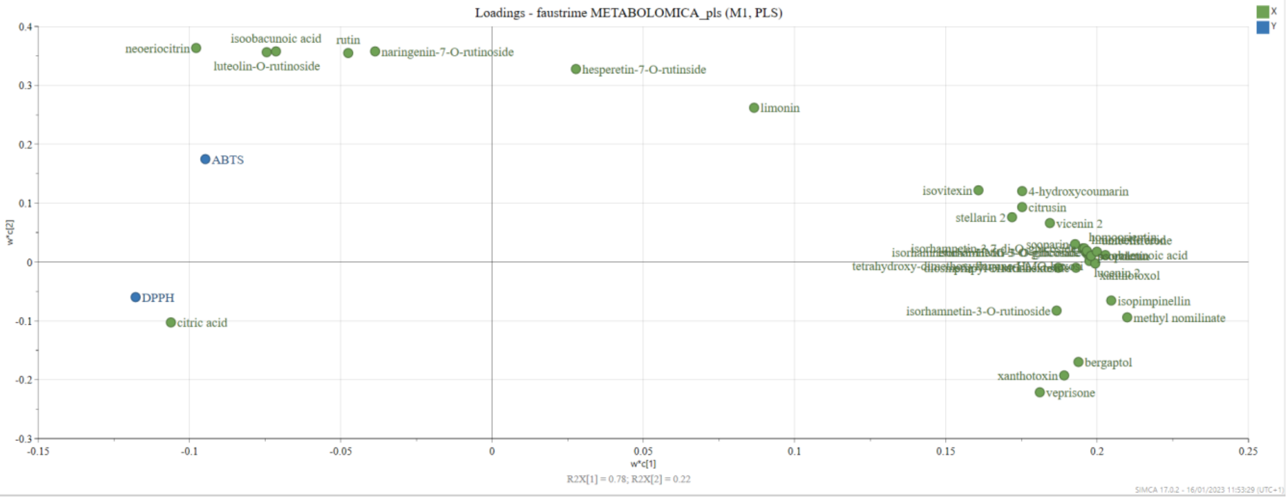

Figure S7. PLS Loading Plot

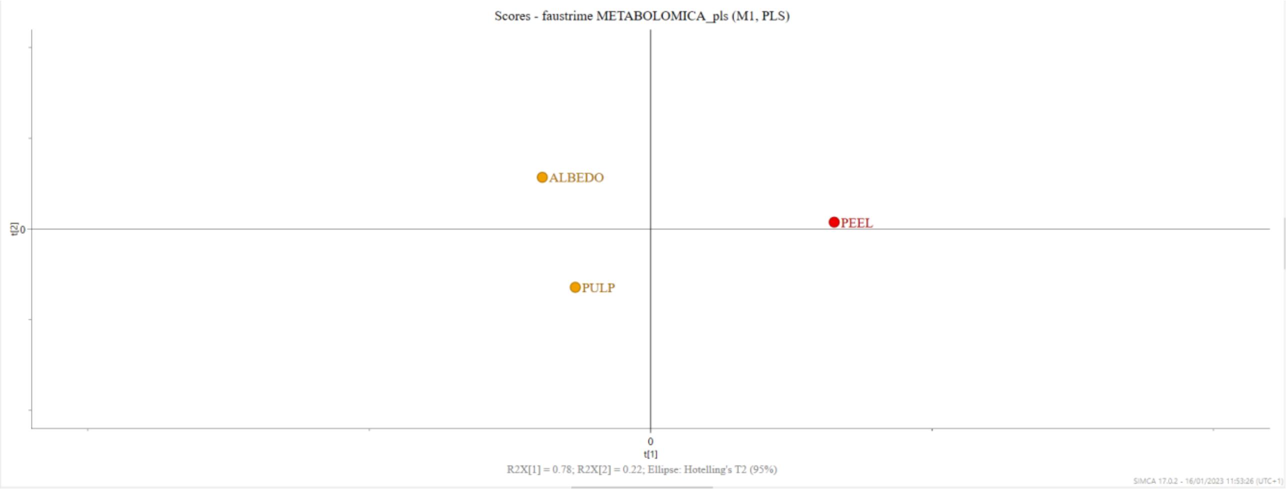

Figure S8. PLS Score Plot

Table S1. Calibration curves for semi-quantitative analysis

**Compounds**	**Linearity (μg mL^{-1})**	**Calibration Equation**	**R^2**
rutin	0.1-50	Y=182.694x - 36.526	0.9997
eriocitrin	0.1-50	Y=428.088x - 20.987	0.9999
quercetin	0.1-50	Y=265.73x – 181.44	0.9969
coumarin	0.1-50	Y=1254.7x - 177.710	0.9999
luteolin	0.1-50	Y=456.827 – 219.854	0.9985
naringenin	0.1-50	Y=754.721x - 99.974	0.9999
apigenin	0.1-50	Y=668.092x - 143.878	0.9999
hesperetin	0.1-50	Y=799.606x – 186.279	0.9997
